# Improving protein-protein interaction prediction using evolutionary information from low-quality MSAs

**DOI:** 10.1371/journal.pone.0169356

**Published:** 2017-02-06

**Authors:** Csilla Várnai, Nikolas S. Burkoff, David L. Wild

**Affiliations:** Systems Biology Centre, University of Warwick, Coventry, CV4 7AL, United Kingdom; University of Michigan, UNITED STATES

## Abstract

Evolutionary information stored in multiple sequence alignments (MSAs) has been used to identify the interaction interface of protein complexes, by measuring either co-conservation or co-mutation of amino acid residues across the interface. Recently, maximum entropy related correlated mutation measures (CMMs) such as direct information, decoupling direct from indirect interactions, have been developed to identify residue pairs interacting across the protein complex interface. These studies have focussed on carefully selected protein complexes with large, good-quality MSAs. In this work, we study protein complexes with a more typical MSA consisting of fewer than 400 sequences, using a set of 79 intramolecular protein complexes. Using a maximum entropy based CMM at the residue level, we develop an interface level CMM score to be used in re-ranking docking decoys. We demonstrate that our interface level CMM score compares favourably to the complementarity trace score, an evolutionary information-based score measuring co-conservation, when combined with the number of interface residues, a knowledge-based potential and the variability score of individual amino acid sites. We also demonstrate, that, since co-mutation and co-complementarity in the MSA contain orthogonal information, the best prediction performance using evolutionary information can be achieved by combining the co-mutation information of the CMM with co-conservation information of a complementarity trace score, predicting a near-native structure as the top prediction for 41% of the dataset. The method presented is not restricted to small MSAs, and will likely improve interface prediction also for complexes with large and good-quality MSAs.

## Introduction

Proteins work together as functional units known as protein complexes to perform the majority of cellular functions, and the analysis of protein-protein interactions forms an essential part of the “systems biology” enterprise. Proteins have to evolve in parallel with their interacting partners, to maintain the functional repertoire of protein complexes. This evolution can be traced by analysing the amino acid sequences of proteins, through the means of multiple sequence alignments (MSAs). Amino acid residues of proteins within protein families exhibit correlations, thus maintaining protein structure and interactions. For example, even when the sequence identity falls to 25%, protein structures typically change by no more than 2 Å [1]. The evolutionary information stored in MSAs has been used in the prediction of protein structure [2–5], stability [6] and interactions [7–17]. In the context of protein-protein interaction prediction, this evolutionary information has been used for the prediction of individual protein interaction sites [7–9] and protein-protein interaction interfaces [10–17].

Amino acid sequence conservation analysis studies [7–10] locating amino acid sites on protein surfaces with low mutation rates can identify protein binding sites. Although it may be possible to test the interface conservation of both binding partners simultaneously with high prediction rates [10], these methods in general are not sensitive to the binding partner. A more advanced conservation method, the SCOTCH complementarity trace method was constructed for the conserved complementarity of protein complex interfaces [11]. This method uses 4 amino acid polarity groups, which mask mutations, enriching the conservation data.

Alternatively, correlated mutation measure (CMM) methods, first pioneered by Valencia and colleagues [12], take a multiple sequence alignment (MSA) profile of evolutionarily-related proteins and attempt to predict residues which have co-evolved. In the context of protein-protein interactions, these methods take into account correlated or compensatory mutations across a protein-protein interface, by construction [13–17]. If residues have co-evolved, this may imply proximity in the native structure or across an interface. For example, if a small residue increases in size by mutating, a proximal residue may have to reduce in size to retain the viability of the fold or complex. Many CMM methods have been developed using Pearson correlation coefficients or other covariance measures, [12–16], adaptations of Mutual Information [18, 19], perturbation methods [20] and Bayesian networks [21]. The disadvantage of using a covariance CMM is that due to the transitive nature of correlation, two amino acids that directly covary only with a third amino acid also appear to vary in a correlated fashion. However, this indirect coupling can be separated out by using the maximum entropy principle, by looking for the least constrained model (containing only the direct coupling between amino acid pairs) that describes the evolutionary constraints on the MSA [22]. One such recently developed correlated mutation measure, the *direct information* (DI) [23, 24], is a global measure which is derived from modelling the entire MSA, specifically defining the probability of each sequence being a member of the MSA. This distribution shares the same low order moments as the MSA, and the maximum entropy principle [25] is used to fully specify the distribution. A number of studies, recently reviewed by Taylor *et al.* [2], have used DI or related measures to successfully aid the structure prediction of a diverse range of proteins of unknown structure [26–34]. However, like the majority of CMM studies, these studies focused on a small number of mainly domain-sized bacterial proteins for which there is a large high quality MSA, because all CM measures suffer as the size of the MSA decreases [35].

Weigt *et al.* [17] also showed that DI can identify interacting amino acid pairs across the interface of a pre-selected protein complex for which a large, good-quality MSA existed. More recently, a number of studies have applied these techniques to the determination of the structures of several protein complexes [36, 37]. As in the case of single protein structure prediction, these studies focused mainly on bacterial protein complexes or others with generally large numbers of sequences in the MSA. However, even with the current advances in genome sequencing, many protein complexes of biological or medical interest have many fewer sequences in their MSA than the large numbers in these studies. For example, 28% of proteins with PFAM IDs [38] predicted to form heterodimer protein-protein interactions by PISA [39] have an effective MSA size (the number of sequences in the MSA divided by the average amino acid sequence length of the interacting proteins) [36, 37] of < 5.

In recent work we have shown, in the protein structure prediction context, that even small auto-generated MSAs of proteins contain useful structural evolutionary information, and that this information can be used to improve the prediction of residue contacts in *β*-sheets, when integrated into a *β*-sheet model [26].

In the present paper, we demonstrate the power of evolution-based information for protein-protein interaction prediction, using a benchmark set of protein complexes from [11], for which the MSAs typically consist of fewer than 400 sequences, with an effective MSA size of 4. A key distinction of this work is that we focus on a wide selection of proteins which have a variety of sizes of MSAs. We also automate the generation of MSAs and do not rely on a high quality MSA being available. As an exemplar of protein complexes, we use the subset of intra-molecular protein complexes, for which better MSAs exist than for inter-molecular complexes. We improve protein complex interface prediction by integrating a maximum entropy based CMM into the scoring function. Instead of focussing at residue level contacts, we define a less noisy interface level CMM score, which we integrate into a scoring function for (FFT-generated) docking decoys. We examine the information stored in the MSA, such as amino acid conservation, co-conservation, and co-mutation, and demonstrate that by combining the information from the maximum entropy based CMM and the SCOTCH complementarity trace analysis [11], the scoring function can be further improved.

## Materials and methods

### The dataset

To benchmark our method, we used the set of intra-molecular protein complexes from [11], using three-fold cross-validation. For each protein complex in the data set, 10,000 docking decoys were generated using the FTDock docking software [40] using the bound protein structures, with the electrostatic correlation score as a binary filter to avoid decoys with unfavourable electrostatic interactions. Only protein complexes with at least one near-native decoy were considered (S1 Table). In our definition, near-native decoys had an interface backbone atom RMSD less than 3 Å from the interface of the native complex, where the interface was defined as all residues interacting with a residue of the other molecule of the complex in the native structure. Two residues were considered interacting if any heavy atom of one residue was within 4.5 Å from any heavy atom of the other residue. The results were robust to the definition of near-native complexes (see Section 1 in S1 Text).

**Fig 1:**
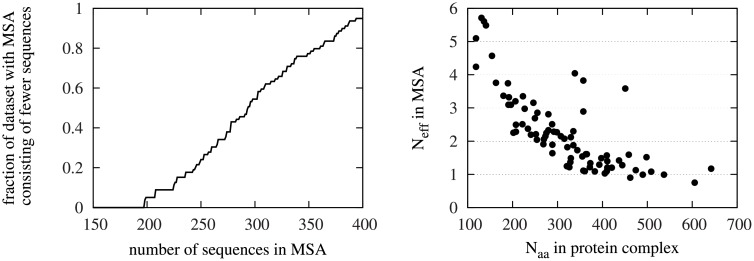
MSAs in the dataset. The cumulative distribution function of protein complexes in the dataset as a function of the number of sequences in their MSA. 95% of protein complexes have fewer than 400 sequences. Right: The effective number of sequences as a function of the number of amino acids in the protein complexes studied.

To be able to investigate the evolutionary information, for each protein complex in our dataset consisting of intra-chromosomal protein complexes, a multiple sequence alignment (MSA) was constructed as in [26] and [41]. For each protein complex, we used the amino acid sequence of the entire multidomain protein, containing the entire sequence of both interacting domains. This way, we end up with paired MSAs by construction, and there is no need to exclude paralogs of the individual domains. Starting from the amino acid sequence, we ran Psi-BLAST [42] for 2 iterations against the nonredundant protein database, keeping all sequences with a sequence identity of at least 30%, as recommended by [43]. Afterwards, the Psi-BLAST alignment was improved and the sequences were trimmed by GLsearch [44] using a global-local alignment algorithm. The trimmed sequences were then clustered with a 98% similarity threshold using cd-hit [45, 46]. The clustered sequences were finally aligned with Muscle [47] yielding the MSA. The MSA was then trimmed to only include the concatenated MSA of the protein complex. The number of sequences in the MSAs of proteins in the dataset varied from 198 to 808 sequences with 95% of the proteins having fewer than 400 sequences in the alignment (Fig 1 Left). The effective number of sequences, i.e. the number of sequences in the alignment divided by the average sequence length of the dimer ([36] and [37]) is shown in Fig 1 Right, and is considerably lower than the number of proteins used by [17] or the effective number of proteins in the MSAs of [36, 37] (S1 Fig).

### Correlated mutation measure

In this work, we used a maximum entropy based CMM that avoids falsely high correlations due to indirect coupling. This CMM is derived as the maximum entropy based CMM we previously developed to improve *β*-sheet contact prediction of proteins [26].

We considered a protein complex with proteins of length *N*_1_ and *N*_2_, with a concatenated MSA containing *M* sequences of length *N* = *N*_1_ + *N*_2_. We defined *f*_*i*_(*A*_*i*_), *i* = 1, …, *N* as the frequency distribution of the amino acid *A*_*i*_ at position *i* of the MSA, and *f*_*ij*_(*A*_*i*_, *A*_*j*_), *i*, *j* = 1, …, *N* as the joint frequency distribution of amino acids *A*_*i*_ and *A*_*j*_ at positions *i* and *j* (*i* and *j* might or might not be on the same protein). Instead of simply looking at the frequency distributions in the MSA of the protein complex, we considered the MSA to be our observation of the underlying concatenated-sequence probability distribution *P*(**A**) of the entire families of the complexed proteins, including observed and unobserved sequences **A** = (*A*_1_, …, *A*_*N*_). We required that our model match the single and pairwise amino acid frequency distributions, *P*_*i*_(*A*_*i*_) = *f*_*i*_(*A*_*i*_) and *P*_*ij*_(*A*_*i*_, *A*_*j*_) = *f*_*ij*_(*A*_*i*_, *A*_*j*_), where *P*_*i*_(.) was the marginal distribution at position *i* and *P*_*ij*_(., .) was the joint marginal distribution at positions *i* and *j*. Any higher-order moments are impractical to consider, due to the sparsity of the data. (The number of possible sequences is *q*^*N*^ ≫ *M*, where *q* is the size of the amino acid library.)

Following the maximum entropy principle, we derived the model distribution that was least biased. The least biased distribution is the one with the highest entropy [48], and variational optimisation resulted in

$$\underset{P(A)}{\mathrm{argmax}}\left[S\left(P\left(A\right)\right)\right]\propto \exp\left(-\sum_{1\leq i<j\leq N}e_{ij}\left(A_{i},A_{j}\right)+\sum_{i=1}^{N}h_{i}\left(A_{i}\right)\right),$$ (1)

where *S*(.) was the entropy of a probability distribution, and *e*_*ij*_ and *h*_*i*_ were the Lagrange multipliers of the constrained optimisation. To perform the optimisation, we used contrastive divergence [26, 49, 50], a statistical machine learning technique. The *e*_*ij*_ can be viewed as pair-interaction energies, and *h*_*i*_ as local fields [17]. In our model of *P*(**A**), the first order moments *P*_*i*_(*A*_*i*_) matched the single amino acid frequencies *f*_*i*_(*A*_*i*_) by construction. The second order moments correspond to the direct correlations via the interaction energies. Our residue-level CMM between residues *i* and *j* was defined as

$$D\left(i,j\right)=\sum_{A_{i},A_{j}}P_{ij}^{D}\left(A_{i},A_{j}\right)\log\frac{P_{ij}^{D}\left(A_{i},A_{j}\right)}{f_{i}\left(A_{i}\right)f_{j}\left(A_{j}\right)}$$ (2)

where

$$P_{ij}^{D}\left(A_{i},A_{j}\right)\propto f_{i}\left(A_{i}\right)f_{j}\left(A_{j}\right)\exp\left(-e_{ij}\left(A_{i},A_{j}\right)\right).$$ (3)

Effectively, $P_{ij}^{D}\left(A_{i},A_{j}\right)$ can be viewed as a probability conditional on the remaining part of the MSA, thus eliminating indirect correlations. This CMM is similar to direct information [17, 23].

For protein interactions, only the joint distributions of amino acid pairs across the interface are of direct interest. However, note that to correctly model the indirect coupling between these amino acid pairs, the model also has to include all pairwise amino acid interactions within the individual proteins.

### The interface level correlated mutation measure score

In the previous section, we described how residue level CMM scores were derived from the concatenated MSA of the complexed proteins. Our CMM, *D*(*i*, *j*), is a measure of the interaction strength between residues *i* and *j*, and high *D*(*i*, *j*) values suggest co-evolution. Based on the residue level CMM, we have developed an interface level CMM score, suitable for ranking the 10,000 decoys generated by FTDock.

First of all, we defined the surface residues to be the amino acids whose relative surface accessibility (the solvent accessibility calculated by WHATIF [51], normalised by the maximal solvent accessibility values [1]) was at least 0.08 on the undocked proteins. The set of surface residues on the same protein as residue *i* were defined as

$$S_{i}=\left\{j:j\;\text{is}\;\text{a}\;\text{surface}\;\text{residue}\;\text{of}\;\text{the}\;\text{same}\;\text{protein}\;\text{as}\;\text{residue}\;i\right\}.$$ (4)

We also defined the set of surface residues on the other protein,

$${\bar{S}}_{i}=\left\{j:j\;\text{is}\;\text{a}\;\text{surface}\;\text{residue}\;\text{of}\;\text{a}\;\text{different}\;\text{protein}\;\text{to}\;\text{residue}\;i\right\}.$$ (5)

**Fig 2:**
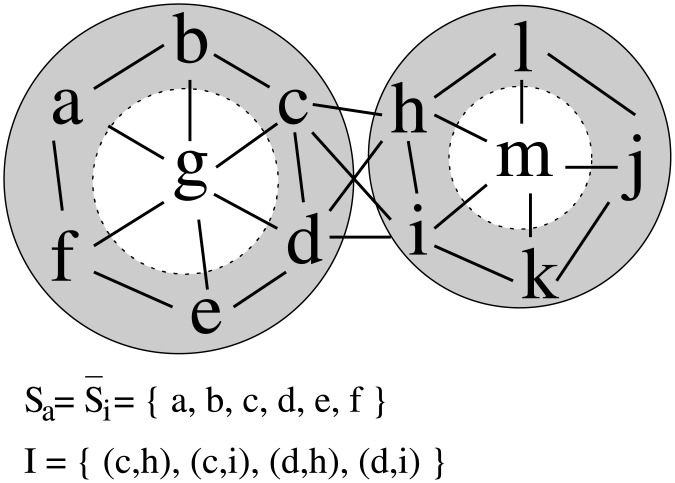
Schematic representation of the protein complex. Solid lines border both proteins, while the surface regions are coloured grey. Interacting amino acids are connected with lines, forming a network. *S*_*a*_ denotes the set of surface residues of the protein that *a* is a residue of, ${\bar{S}}_{i}$ denotes the set of surface residues of the protein that *i* is not a residue of, and *I* denotes the set of interactions across the interface.

The set of interface interactions, I, in the protein complex was

$$I=\left\{\left(i,j\right):j\in {\bar{S}}_{i},i\in {\bar{S}}_{j},\;\text{and}\;d_{ij}<4.5Å\right\}$$ (6)

where *d*_*ij*_, the distance between residues *i* and *j*, was the smallest distance between any heavy atom of residue *i* to any heavy atom of residue *j*. We defined

$$N_{i}=\left\{\begin{array}{cc} \left\{j:j\in S_{i}\;\text{and}\;d_{ij}<4.5Å\right\} & \;\text{if}\;i\in S_{i}\\ S_{i} & \;\text{if}\;i\notin S_{i} \end{array}\right.$$ (7)

as the surface neighbourhood of a surface residue *i*, or all surface residues on the protein for non-surface residues. For example, in Fig 2, $S_{a}={\bar{S}}_{i}=\{a,b,c,d,e,f\}$, N(c)={b,c,d} and N(j)={j,k,l}.

We defined the interface-level raw CMM score as

$$S_{\text{raw}}^{\text{CMM}}=\frac{1}{\left|I\right|}\sum_{(i,j)\in I}D\left(i,j\right).$$ (8)

However, as protein structures are flexible and amino acid side chains may move about, the co-evolution of a surface neighbour of residue *j* with the potential interaction partner $i\in {\bar{S}}_{j}$ may also imply a contact between *i* and *j*. This idea has been used previously for protein structure [26] and protein-protein interaction prediction [11]. In this work, we defined an interface level CMM score, based on neighbour-averaged residue-level CMM scores. The neighbour-averaged CMM score of residue *i* was defined as

$$\chi \left(i,j\right)=\frac{1}{\left|N_{j}\right|}\sum_{k\in N_{j}}D\left(i,k\right).$$ (9)

However, for different residues *i*, the mean and variance of the set of values $\left\{D\left(i,k\right):k\in N_{j}\right\}$ differed wildly and so *χ*(*i*, *j*) needed to be standardised before being used. For this standardisation we took the sample mean *μ*_*i*_ and standard deviation *σ*_*i*_ of the set $\left\{\chi \left(l,k\right):l\in S_{i},\left(l,k\right)\in I\right\}$ [26]. We symmetrised the standardized scores, yielding

$$Z\left(i,j\right)=Z\left(j,i\right)=\frac{1}{2}\left(\frac{\chi \left(i,j\right)-\mu_{i}}{\sigma_{i}}+\frac{\chi \left(j,i\right)-\mu_{j}}{\sigma_{j}}\right),$$ (10)

the symmetrised neighbour-averaged CMM scores for residue pairs (i,j)∈I. Finally, we averaged this standardised score over the interface interactions to obtain the interface-level neighbour-averaged CMM score,

$$S^{\text{CMM}}\left(I\right)=\frac{1}{\left|I\right|}\sum_{i,j\in I}Z\left(i,j\right).$$ (11)

### The combined scoring function

We have integrated the *S*^CMM^ score into a combined scoring function which includes structural as well as evolutionary information, in a similar manner to previous studies [11, 52]. The scoring function, *S*, was chosen to be the logit function of the likelihood of the decoy being a near native structure, taking a positive value for decoys that are more likely to be native than non-native. We defined the combined scoring function as

$$S=w_{0}+w^{N}S^{N}+w^{\text{RP}}S^{\text{RP}}+w^{\text{ent}}S^{\text{ent}}+w^{\text{CT}}S^{\text{CT}}+w^{\text{CMM}}S^{\text{CMM}}$$ (12)

where the *S*’s were scores described below and the *w*’s were weights to be optimised. *S*^N^ was the number of interface residues of the docking decoy. *S*^RP^ was a knowledge based potential, the Residue Pair potential (RP) score [53], as implemented in the FTDOCK distribution [40]. *S*^ent^ was the entropy score, the average of the entropy in the MSA for the individual interface residues (−∑_*a*_ *P*(*a*) ln *P*(*a*)) weighted by the number of residue interactions across the interface. *S*^CT^ was the Complementarity Trace (CT) score, quantifying the proportion of interacting interface residue pairs for which complementarity was preserved in the MSA, adapted from [11]. All residues were categorised into 4 groups (hydrophobic, polar, positively charged and negatively charged), and complementarity required two residues to be both hydrophobic, both polar or having opposite charges. A residue pair across the interface was defined to be complementary, if one of the residues was complementary with the other residue or its first or second structural neighbours, when projecting the residues onto the docked structure. Residues of the same molecule were considered to be structural neighbours if they had any heavy atoms within 4.5 Å. The complementarity was considered preserved in the MSA, if the residue pair was complementary in 95% of the sequences of the MSA. *S*^CMM^ is the Correlated Mutation Measure (CMM) score described in the previous section.

The following forms of the scoring function were considered in this work:

$$\begin{array}{c} S^{\text{CMM}}\\ S\left(S^{N},S^{\text{RP}},S^{\text{ent}}\right)=w_{0}+w^{N}S^{N}+w^{\text{RP}}S^{\text{RP}}+w^{\text{ent}}S^{\text{ent}}\\ S\left(S^{N},S^{\text{RP}},S^{\text{ent}},S^{\text{CMM}}\right)=w_{0}+w^{N}S^{N}+w^{\text{RP}}S^{\text{RP}}+w^{\text{ent}}S^{\text{ent}}w^{\text{CMM}}S^{\text{CMM}}\\ S\left(S^{N},S^{\text{RP}},S^{\text{ent}},S^{\text{CT}}\right)=w_{0}+w^{N}S^{N}+w^{\text{RP}}S^{\text{RP}}+w^{\text{ent}}S^{\text{ent}}+w^{\text{CT}}S^{\text{CT}}\\ S\left(S^{N},S^{\text{RP}},S^{\text{ent}},S^{\text{CMM}},S^{\text{CT}}\right)=w_{0}+w^{N}S^{N}+w^{\text{RP}}S^{\text{RP}}+w^{\text{ent}}S^{\text{ent}}+w^{\text{CT}}S^{\text{CT}}+w^{\text{CMM}}S^{\text{CMM}} \end{array}$$ (13)

The scoring function was optimised using logistic regression by the generalised linear model function (glm) of R, using the binomial family and logit as the link function. Results were obtained using three-fold cross-validation: two thirds of the data set were used as the training set for the optimisation, and the remaining one third as the validation set (S1 Table).

## Results and discussion

### Residue level CMM scores are noisy contact predictors

**Fig 3:**
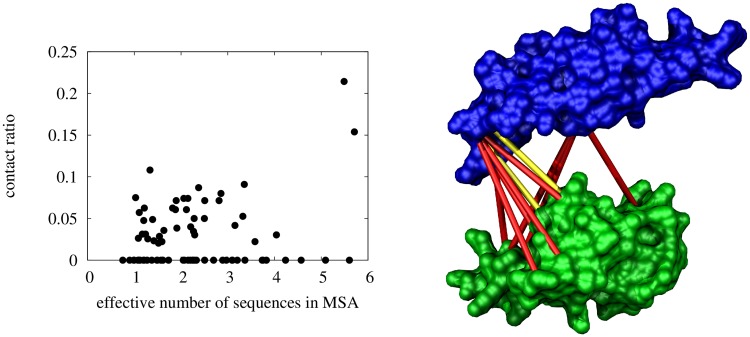
Residue-level CMM scores are noisy interface predictors. Left: The proportion of true contacts in the top *N*/10 *Z*(*i*, *j*) scores. Right: The top 10 contacts predicted by the residue level CMM scores for D1FJGE1_D1FJGE2, the protein complex with the highest contact ratio (0.21). True contacts are coloured yellow, false contacts are coloured red.

We first investigated if the residue level standardised CMM scores could be used as predictors of inter-protein residue contacts, for our data set with small auto-generated MSAs. The effective number of sequences in our data set was typically much smaller than in other studies [36, 37], as shown in S1 Fig. We found that for 6 of the 79 protein complexes the top CMM score corresponded to a true contact. However, these contacts were corroborated by no or very few other contacts (for any protein, the 5 highest CMM scores would only have no more than 1 true contact). In general, for all protein complexes fewer than a quarter of the top N/10 predictions were true contacts, where N is the combined residue length of the complex (Fig 3). Fig 3 Left shows the proportion of true contacts (contact ratio) in the top N/10 predicted contacts for all protein complexes in the data set. The high false positive rate is also illustrated in Fig 3 Right, depicting the top 10 residue level CMM scores as contacts predicted for D1FJGE1_D1FJGE2, the protein complex with the highest contact ratio (0.21 on Fig 3 Left). Predictions by the GREMLIN [36, 54] and EVCOMPLEX [37, 55] webservers were similarly unreliable (S2 Table).

**Fig 4:**
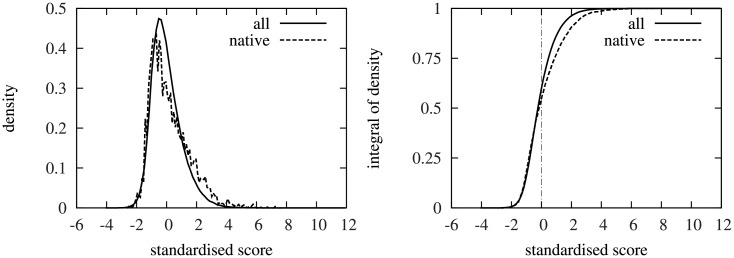
Probability distribution of the residue-level CMM scores. The distribution of the standardised *Z*(*i*, *j*) scores for all residues (solid line) and for the interface residues of the native structure (dashed line). Left: Probability distribution function, Right: cumulative distribution function. Dash-dotted line shows 0, the mean of the standardised scores.

To quantify the amount of information the CMM scores contain about the native interactions, we calculated the distribution function of the residue-level standardised CMM scores for the natively interacting and all inter-protein surface-residue pairs. We found that although both the native and all inter-protein residue-level CMM scores had a heavy tail in the positive direction, the distribution function of the residue-level standardised CMM scores had a significantly larger probability density in the positive domain for native interface residue contacts than for all inter-protein surface residue pairs (Fig 4). These results suggested that although individual residue-level CMM scores were noisy contact predictors, integrating the residue-level CMM scores into interface-level CMM scores might improve the signal-to-noise ratio, and hence the interaction interface prediction accuracy.

### Interface level CMM scores improve interaction site prediction accuracy

**Fig 5:**
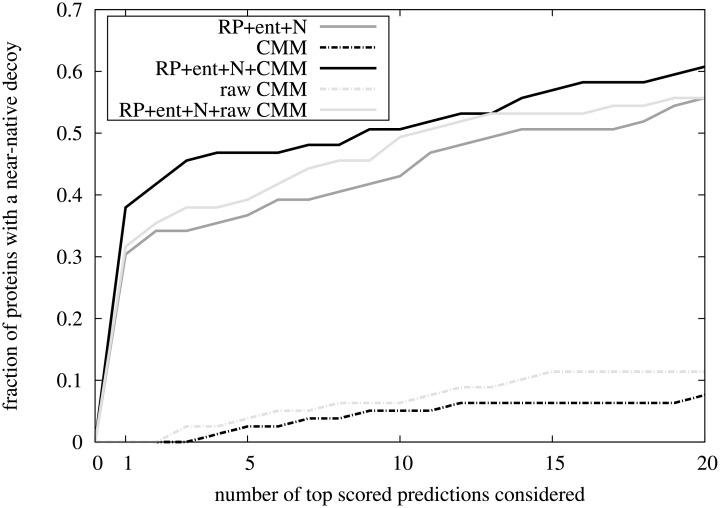
Comparison of the interface-level scoring functions using CMM. The fraction of proteins for which there is at least one near-native complex in the top predictions, for the scoring functions *S*^CMM^ (black dash-dotted line), $S_{\text{raw}}^{\text{CMM}}$ (light grey dash-dotted line), *S*(*S*^RP^, *S*^N^, *S*^ent^) (grey solid line), *S*(*S*^RP^, *S*^N^, *S*^ent^, *S*^CMM^) (black solid line) and $S(S^{\text{RP}},S^{N},S^{\text{ent}},S_{\text{raw}}^{\text{CMM}})$ (light grey solid line).

Next, we evaluated the effect of our interface-level CMM scores on the protein-protein interaction interface prediction accuracy. We used the raw interface-level CMM scores (Eq 8), the surface-neighbour-averaged interface-level CMM scores (Eq 11), and the combined scoring functions *S*(*S*^RP^, *S*^N^, *S*^ent^), *S*(*S*^RP^, *S*^N^, *S*^ent^, *S*^CMM^) and $S(S^{\text{RP}},S^{N},S^{\text{ent}},S_{\text{raw}}^{\text{CMM}})$ (Eq 13), to rank the complex decoys generated by FTDock. The fraction of protein complexes in the data set with a near-native prediction in the top predictions as a function of the number of top predictions considered is shown in Fig 5. Although the interface-level CMM scores themselves were poor complex interface predictors (no near-native docking decoys were found in the top 2 predictions), integrating the surface-neighbour-averaged interface-level CMM score into a combined score with the number of interface residues plus a knowledge based potential and the entropy score, the interface prediction improved significantly. Using the combined score *S*(*S*^RP^, *S*^N^, *S*^ent^, *S*^CMM^), 6 more complexes (7.6% of the dataset) had a near-native prediction in the top 1 predictions than without the *S*^CMM^ score, indicating that even for protein complexes without a large, good-quality MSA, the CMM scores can be used to improve interface prediction accuracy.

We next focussed on the effect of surface-neighbour averaging of the CMM scores. Although the interface-level raw CMM score was a somewhat better predictor in itself than the surface-neighbour-averaged interface-level CMM score, incorporating it into the combined score gave less improvement on the prediction. When examining the list of protein complexes with near-native decoys in the top 20 predictions, out of the 9 and 6 proteins for the raw and surface-neighbour-averaged CMM scores, there was only 1 protein complex for which both CMM scores had a near-native prediction (S1 Table). The reason for this small overlap was that by averaging the *D*(*i*, *j*) scores over surface neighbours, the (native or non-native) strong residue-level *D*(*i*, *j*) scores were diluted. On the one hand, for protein complexes with few strong natively interacting residue-level *D*(*i*, *j*) scores, the surface-neighbour averaging resulted in poorer interface prediction. On the other hand, for some protein complexes, where a few non-native residue-level *D*(*i*, *j*) scores obliterated the low but consistently more positive native residue-level *D*(*i*, *j*) scores (see previous section), this averaging increased the signal-to-noise ratio. Using the less noisy *S*^CMM^ then resulted in a more marked improvement when incorporated into a combined score. These results are in agreement with previous observations that evolutionary information contributions from surface residues close to the interface might well play a role in the assembly of protein complexes [11, 56–58].

### Combining evolutionary information from conservation and co-mutation gives best prediction results

Next, we considered what evolutionary information could be extracted from the MSA. The first order moments of the MSA describe the distributions of individual amino acid sites, and their variability is measured by the entropy score. The second moments contain the pairwise amino acid site distributions, which can be modelled by looking at co-mutations or co-conservation. By definition, the CMM models co-mutating residue pairs, and it does not interrogate the co-conservation of pairs of amino acid residues across the interface. Co-conservation information, on the other hand, does not measure co-mutations, although some co-mutation information may be recovered by collapsing the 20 amino acid residues into groups of residues, as in the SCOTCH complementarity trace (CT) score [11]. There has been little comparison of correlated mutation and conservation information [11]. In this work, we compared the effects of the CMM and CT scores on the prediction accuracy in more detail, and, unlike previous studies, we extended this comparison to include a combined scoring function containing both the CT and CMM scores.

**Fig 6:**
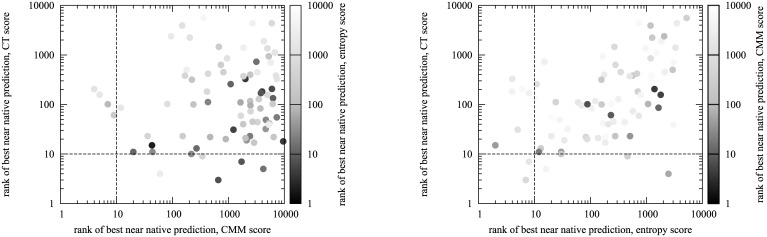
Comparison of co-conservation and co-evolution scores. Left: Scatter plot showing the rank of the best near-native prediction for the CMM (horizontal axis) against the CT (vertical axis) score (*R*_Pearson_ = −0.02), coloured by the rank of the best near-native prediction of the entropy score. Right: Scatter plot showing the rank of the best near-native prediction for the entropy (horizontal axis) against the CT (vertical axis) score (*R*_Pearson_ = 0.60), coloured by the rank of the best near-native prediction of the CMM score.

We compared the best near-native predictions of the individual CT, CMM and entropy scores (Fig 6). There were 6, 4 and 10 protein complexes, for which the individual CT, CMM and entropy scores had a near-native decoy in the 10 highest scoring predictions, with 2 complexes predicted correctly by both the CT and entropy scores. There was no overlap between the protein complexes predicted correctly by the CMM score and either the CT or the entropy score. Moreover, while there was no correlation between the rank of best near-native prediction for the CMM and the entropy scores (Pearson’s *R* = −0.02), there was a significant correlation between the entropy and CT score predictions (Pearson’s R = 0.60). These results reflected the theoretical considerations about the information stored in the MSA measured by the three scores. On the one hand, we found that for protein complexes with plenty of strongly co-mutating residue pairs, where the CMM signal was strong, the entropy score was a weak predictor, and the CT score, using an amino acid alphabet reduced to 4 polarity groups, ignored the more subtle changes in co-mutations. Mintseris *et al.* [59] had found that the maximum information could be extracted when using an alphabet size of 12, however, for small MSAs, a reduced alphabet size would be necessary. On the other hand, for protein complexes with an interface across which residue pairs rarely mutated, we found that both the entropy and CT scores were high but the CMM signal was very weak; this was especially true for protein complexes with smaller MSAs. These results demonstrate that including information from both co-mutation and co-conservation information derived from the second order moments of the MSA offers the best prediction performance.

**Fig 7:**
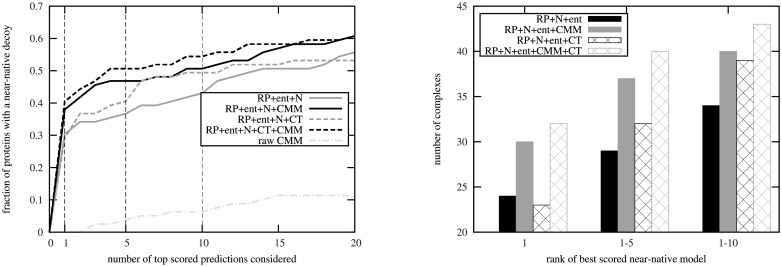
The effect of co-conservation and co-evolution on the interface prediction. Left: The fraction of proteins for which a near-native decoy is in the top scored predictions, as a function of the number of decoys considered, for the *S*(*S*^RP^, *S*^N^, *S*^ent^) (grey solid line), *S*(*S*^RP^, *S*^N^, *S*^ent^, *S*^CMM^ (black solid line), *S*(*S*^RP^, *S*^N^, *S*^ent^, *S*^CT^ (grey dashed line), *S*(*S*^RP^, *S*^N^, *S*^ent^, *S*^CT^, *S*^CMM^) (black dashed line) and $S_{\text{raw}}^{\text{CMM}}$ (light grey dash-dotted line) scoring functions. Right: The number of proteins for which the rank of the top near-native prediction is within the top 1, 5 or 10 predictions, for the *S*(*S*^RP^, *S*^N^, *S*^ent^) (solid black bars), *S*(*S*^RP^, *S*^N^, *S*^ent^, *S*^CMM^ (solid grey bars), *S*(*S*^RP^, *S*^N^, *S*^ent^, *S*^CT^ (dark checked bars) and *S*(*S*^RP^, *S*^N^, *S*^ent^, *S*^CT^, *S*^CMM^) (light checked bars) scoring functions.

We then compared the fraction of proteins with a near-native decoy in the top predictions for combined scoring functions containing the CT, the CMM, neither and both scores (Fig 7). When incorporating the CT score rather than the CMM score into the combined scoring function, *S*(*S*^ent^, *S*^N^, *S*^RP^, *S*^CT^), there was a smaller improvement in the prediction accuracy. In the top 1 prediction, the combined score including the CT score predicted 1 fewer protein complexes correctly than without the CT score, while the combined score including the CMM score predicted 6 more protein complexes correctly in the top 1 predictions. Incorporating both the CMM and the CT scores into a combined score provides the best prediction of near-native complexes, as, compared to the score with neither the CMM nor the CT scores, it gave a near-native prediction for 8 more protein complexes (10% of the dataset) in the top prediction (for some examples, see S2, S3 and S4 Figs). This demonstrates that by combining the two approaches, we can extract more information from the MSA, further improving the prediction performance. This improvement would then allow for the reduction of the number of docking decoys that needed to be considered in the further computationally intensive refinement of the docked complexes [60].

## Conclusion

We have demonstrated the predictive power of a maximum entropy-based correlated mutation measure for protein complex interaction interfaces, using proteins for which only small, auto-generated multiple sequence alignments exist. As an exemplar of protein-protein interaction complexes, we have used a data set of intramolecular complexes with typically fewer than 400 sequences in the MSA, and calculated a maximum entropy-based CMM score representing the strength of direct amino acid residue interactions within the protein complex. In this study, we studied the interface prediction for intra-molecular complexes, as this eliminates further noise from possible paralogs in the MSA. We also generated docking decoys from the bound conformation of proteins, consistent with the original study presenting the CT score [11]. It is the scope of further study to extend the analysis to inter-chromosomal complexes and docking from unbound structures.

For proteins with large good-quality MSAs, individual CMM scores could be used for protein-protein interaction interface prediction (for example, [17]). In contrast, for the protein complexes with small MSAs studied here, the residue-level CMM measure in itself is a very noisy predictor of interacting amino acid residues across the interaction interface, using the method presented here as well as for other CMM based methods (GREMLIN [36, 54] and EVCOMPLEX [37, 55]). We present an interaction interface level patch score calculated from neighbour-averaged standardised residue-level CMM scores, and integrate it into a scoring function function with other predictors, such as the number of interactions across the interface, a knowledge-based potential, and a residue-level variability score in the MSA, following [11].

We demonstrate that the interface level CMM score presented here improves the interface prediction, and it does that by by adding orthogonal information. First of all, the maximum entropy-based CMM measures the direct correlation of co-mutating amino acid residue pairs across the interface, decoupled from indirect correlations. Secondly, by performing a surface-neighbour averaging in the calculation of interface level patch scores, amino acid residues neighbouring the interface, but not directly involved in interactions in the rigid complex can also contribute their direct correlation information to the interface residues of the other protein.

We have investigated the evolutionary information stored in the MSA, by comparing co-conservation (*S*^ent^, *S*^CT^) and co-mutation (*S*^CMM^) prediction scores based on the first and second-order moments of the MSA. We find that the co-conservation scores of the first- and second-order moments were correlated, and from the second-order moments, overall a combined score using the CMM (*S*(*S*^RP^, *S*^N^, *S*^ent^, *S*^CMM^)) performed better than the CT score (*S*(*S*^RP^, *S*^N^, *S*^ent^, *S*^CT^)) for the studied dataset. We also find that a combined score including both the CMM and complementarity trace scores (*S*(*S*^RP^, *S*^N^, *S*^ent^, *S*^CT^, *S*^CMM^)) had the best predictive performance, as co-mutation and co-conservation represent complementary information. We note that in other datasets with more amino acid conservation at the interface, the co-conservation score may have stronger prediction power than co-mutation, as is suggested by our findings that different protein complexes were predicted correctly by the co-conservation and co-mutation based combined scores. However, the complementarity of information captured by the co-conservation and co-mutation scores would still hold. Our results demonstrate that the best strategy for protein-protein interaction interface prediction is to combine co-mutation and co-conservation in a joint scoring function. We suggest that our results are not limited to protein complexes with small MSAs, and a joint scoring function would improve protein-protein interaction interface prediction for complexes with larger, better-quality MSAs.

## Supporting information

S1 Text Robustness of results to the complex RMSD definition. (PDF)

S1 Fig The effective number of sequences in the data set compared to other studies. The effective number of sequences as a function of the average length of the proteins in the complexes. Blue dots show the data set used in [36], red dots show the data set used in [37], and black dots show the data set used in this study.
(PDF)

S2 Fig Prediction improvement for D1A5KC1_D1A5KC2 using the combined score. The interaction scores plotted against the interface level RMSD, for the D1A5KC1_D1A5KC2 complex. Vertical dashed line shows the near-nativeness threshold (RMSD<3Å). The scoring functions used are the same as in Fig 7 Right. The addition of the CMM score brings the top-predicted near-native complex from 79th (RP+N+ent) to 4th place (RP+N+ent+CMM and RP+N+ent+CMM+CT, also see Table S2 Table).
(PDF)

S3 Fig Prediction improvement for D1AY0A2_D1AY0A3 using the combined score. The interaction scores plotted against the interface level RMSD, for the D1AY0A2_D1AY0A3 complex. Vertical dashed line shows the near-nativeness threshold (RMSD<3Å). The scoring functions used are the same as in Fig 7 Right. The addition of either the CMM or the CT score brings the top-predicted near-native complex from 2nd to 1st place (also see Table S2 Table).
(PDF)

S4 Fig Prediction improvement for D1DTWB1_D1DTWB2 using the combined score. The interaction scores plotted against the interface level RMSD, for the D1DTWB1_D1DTWB2 complex. Vertical dashed line shows the near-nativeness threshold (RMSD<3Å). The scoring functions used are the same as in Fig 7 Right. The addition of the CMM score brings the top-predicted near-native complex from 19th to 1st place (also see Table S2 Table).
(PDF)

S1 Table The protein complexes. The list of protein complexes in the data set, with the number of amino acid residues in the complex (N), the number of sequences in the MSA (M), the top near native complex prediction for the different scoring functions, and the test set the protein complexes belonged to.
(PDF)

S2 Table GREMLIN and EVCOMPLEX predictions. GREMLIN and EVCOMPLEX predictions for the protein complexes in the data set. For each complex, the number of sequences in the MSA relative to the number of residues in the complex (M/N) and the rank of the top predicted native contact for both methods are shown. In the M/N columns, ‘—’ means no predictions due to not enough sequences (GREMLIN) or failed concatenation (EVCOMPLEX).
(PDF)
