# Supplementary material for: Improving protein-protein interaction prediction using evolutionary information from low-quality MSAs
Source: PLoS One. 2017 Feb 6;12(2):e0169356. doi: 10.1371/journal.pone.0169356 (PMC5293240; doi:10.1371/journal.pone.0169356)
Supplement: S1 Text — (PDF) [file pone.0169356.s001.pdf]

S1 Text. Robustness with respect to the definition of near-native structures

We tested if the improvement of the prediction performance was robust to the definition of near-nativeness. As an alternative definition of a near-native complex, we used the RMSD of the backbone heavy atoms of all native interface residues i, for which $(i, j) \in \mathcal{I}$ for some other interface residue j. A complex was defined near-native if this RMSD < 3.0 Å. This interface residue RMSD and the ligand interface residue RMSD after aligning the receptor protein showed strong correlation. Figure 1 of S1 Text the correlation for the D1A04A1_D1A04A2 protein complex (Pearson $R = 0.98$). Using the interface residue RMSD definition, the number of near-native complexes in the three data sets was slightly lower than when the ligand interface residue RMSD was used (Table 1 of S1 Text). However, the success curves do not change significantly with the different RMSD definition (Figure 2 of S1 Text).

data set	int-RMSD	Lint-RMSD
joint A+B	251	275
joint A+C	187	263
joint B+C	301	335

Table 1 of S1 Text : The number of near-native protein complexes identified in the data set, for the interface residue RMSD (int-RMSD) and for the ligand interface residue RMSD after aligning the receptor protein (Lint-RMSD).

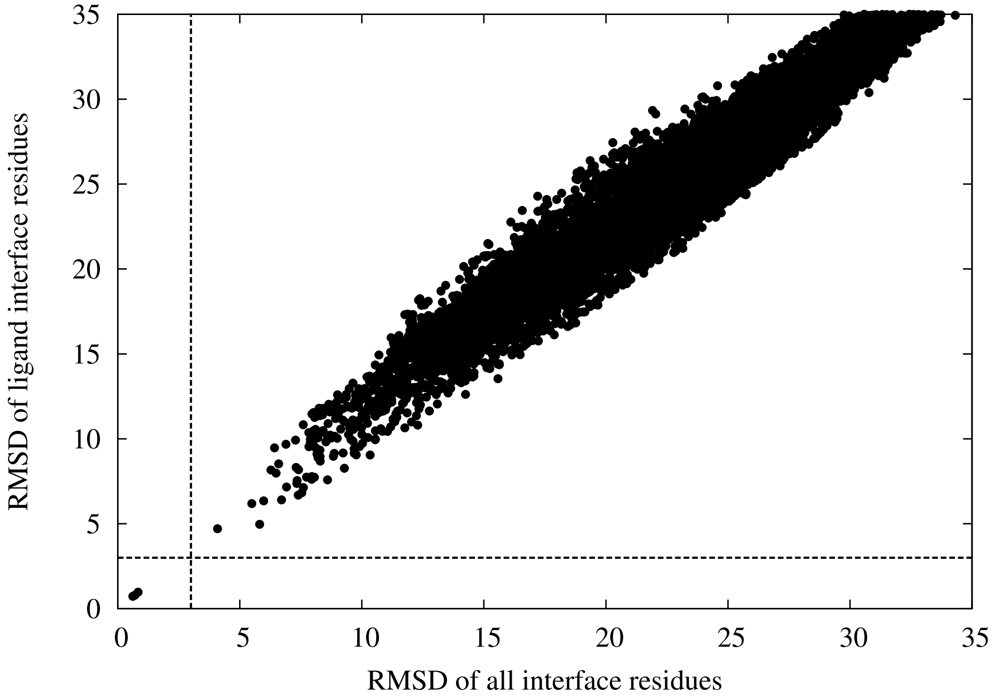

Figure 1 of S1 Text : Correlation between the backbone heavy atom RMSD of ligand interface residues after the alignment of the receptor protein (horizontal axis, used in the main text) and the backbone heavy atom RMSD of all interface residues (vertical axis). Complex: D1A04A1_D1A04A2. Pearson's correlation: 0.98. Dashed lines indicate the threshold of near-nativeness (3 Å).

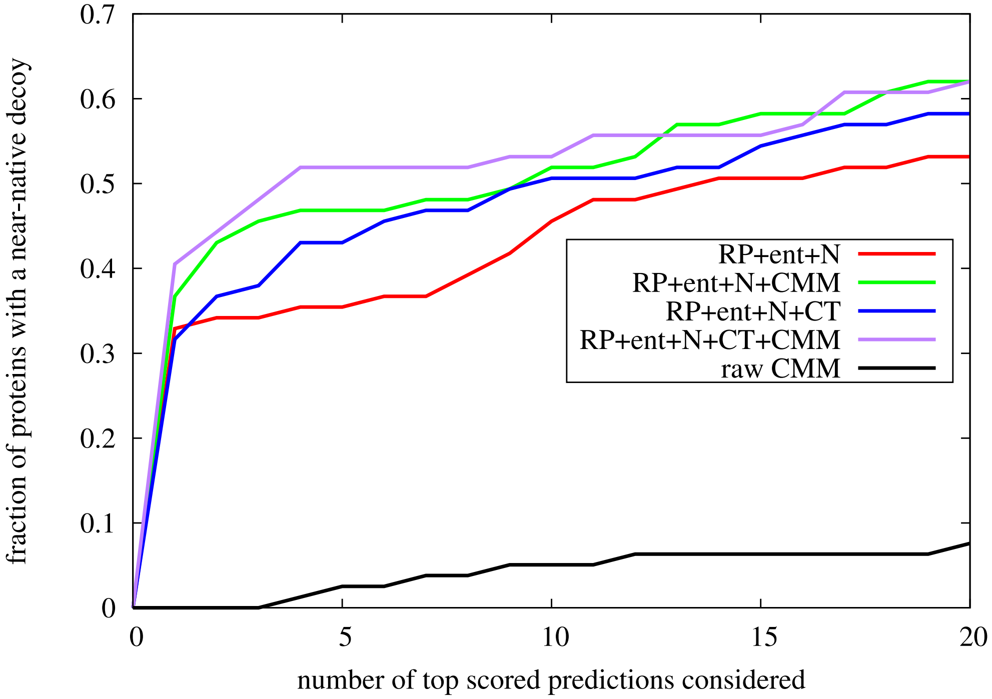

Figure 2 of S1 Text : The same figure as Figure 7 in the Main Text, generated using the interface residue RMSD. The scoring functions are $S^{\rm CMM}$ (black), $S(S^{\rm RP}, S^{\rm N}, S^{\rm ent})$ (red), and $S(S^{\rm RP}, S^{\rm N}, S^{\rm ent}, S^{\rm CMM})$ (green), $S(S^{\rm RP}, S^{\rm N}, S^{\rm ent}, S^{\rm CT})$ (blue), $S(S^{\rm RP}, S^{\rm N}, S^{\rm ent}, S^{\rm CT}, S^{\rm CMM})$ (purple).
