# Supplementary figures and images for: Improving protein-protein interaction prediction using evolutionary information from low-quality MSAs
Source: PLoS One. 2017 Feb 6;12(2):e0169356. doi: 10.1371/journal.pone.0169356 (PMC5293240; doi:10.1371/journal.pone.0169356)

$N_{\text{eff}}$  in MSA

this study  
Ovchinnikov et al.  
Hopf et al.

●  
●  
●

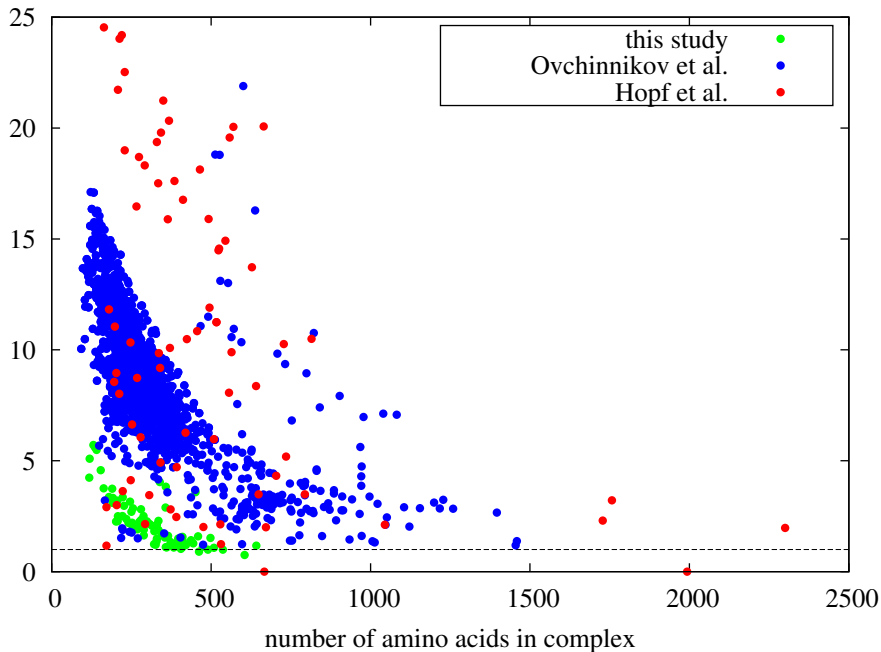

Supplement: S1 Fig — The effective number of sequences as a function of the average length of the proteins in the complexes. Blue dots show the data set used in [36], red dots show the data set used in [37], and black dots show the data set used in this study. (PDF) [file pone.0169356.s002.pdf]

no CMM

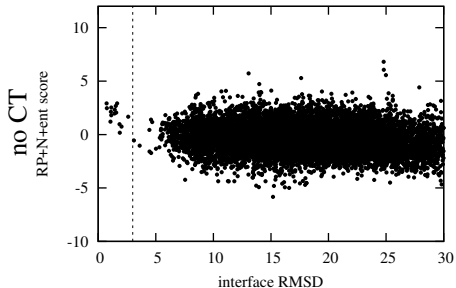

with CMM

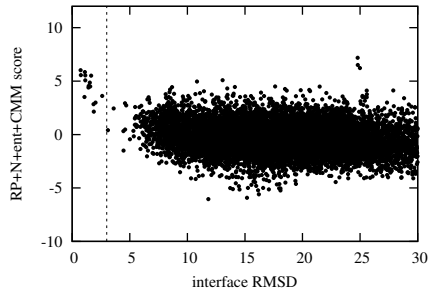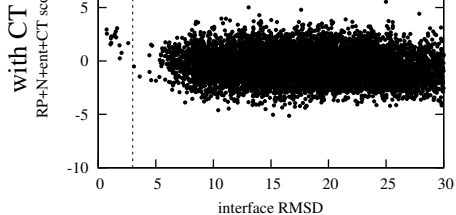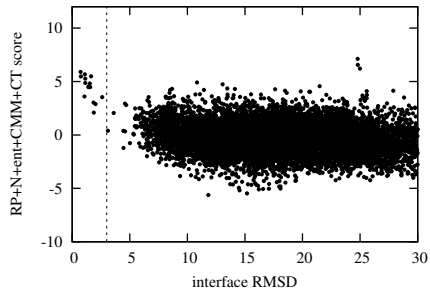

Supplement: S2 Fig — The interaction scores plotted against the interface level RMSD, for the D1A5KC1_D1A5KC2 complex. Vertical dashed line shows the near-nativeness threshold (RMSD<3Å). The scoring functions used are the same as in Fig 7 Right. The addition of the CMM score brings the top-predicted near-native complex from 79th (RP+N+ent) to 4th place (RP+N+ent+CMM and RP+N+ent+CMM+CT, also see Table S2 Table). (PDF) [file pone.0169356.s003.pdf]

no CMM

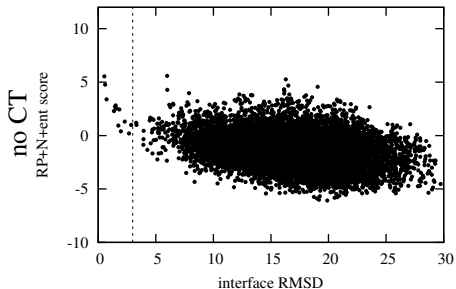

with CMM

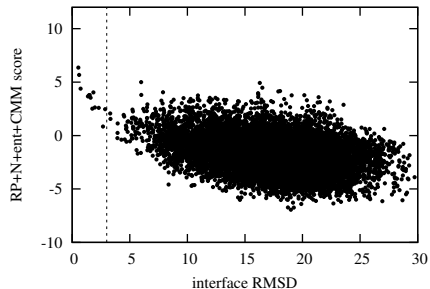

with CT

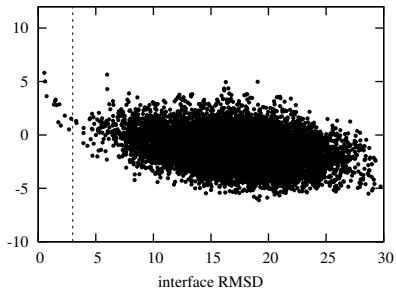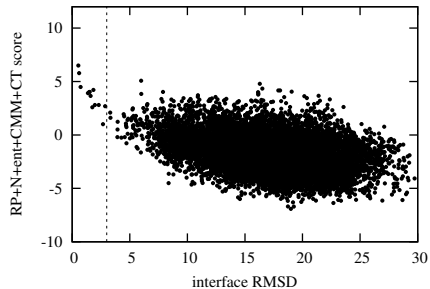

Supplement: S3 Fig — The interaction scores plotted against the interface level RMSD, for the D1AY0A2_D1AY0A3 complex. Vertical dashed line shows the near-nativeness threshold (RMSD<3Å). The scoring functions used are the same as in Fig 7 Right. The addition of either the CMM or the CT score brings the top-predicted near-native complex from 2nd to 1st place (also see Table S2 Table). (PDF) [file pone.0169356.s004.pdf]

no CMM

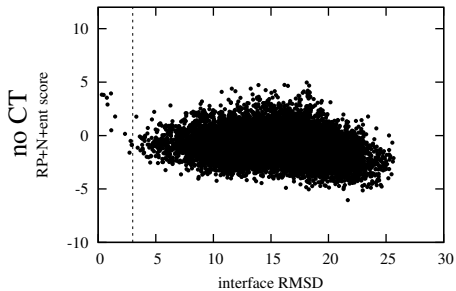

with CMM

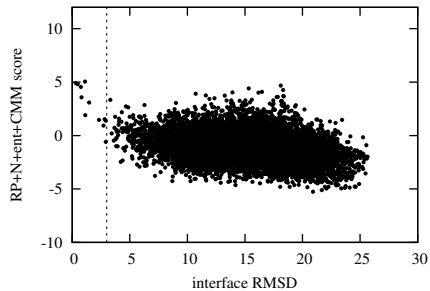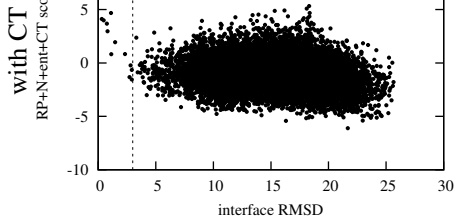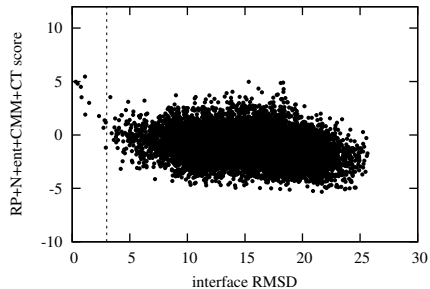

Supplement: S4 Fig — The interaction scores plotted against the interface level RMSD, for the D1DTWB1_D1DTWB2 complex. Vertical dashed line shows the near-nativeness threshold (RMSD<3Å). The scoring functions used are the same as in Fig 7 Right. The addition of the CMM score brings the top-predicted near-native complex from 19th to 1st place (also see Table S2 Table). (PDF) [file pone.0169356.s005.pdf]
