# Supplementary material for: Improving protein-protein interaction prediction using evolutionary information from low-quality MSAs
Source: PLoS One. 2017 Feb 6;12(2):e0169356. doi: 10.1371/journal.pone.0169356 (PMC5293240; doi:10.1371/journal.pone.0169356)
Supplement: S1 Table — The list of protein complexes in the data set, with the number of amino acid residues in the complex (N), the number of sequences in the MSA (M), the top near native complex prediction for the different scoring functions, and the test set the protein complexes belonged to. (PDF) [file pone.0169356.s006.pdf]

complex	N	M	$S^{\mathrm{CMM}}_{\mathrm{raw}}$	S^{CMM}	$S(S^{\mathrm{RP}}, S^{\mathrm{N}}, S^{\mathrm{ent}})$ +ent	$S(S^{\mathrm{RP}}, S^{\mathrm{N}}, S^{\mathrm{ent}}, S^{\mathrm{CMM}})$	$S(S^{\mathrm{RP}}, S^{\mathrm{N}}, S^{\mathrm{ent}}, S^{\mathrm{CT}})$	$S(S^{\mathrm{RP}}, S^{\mathrm{N}}, S^{\mathrm{ent}}, S^{\mathrm{CT}}, S^{\mathrm{CMM}})$	test set
D1A04A1_D1A04A2	180	303	2934	3293	225	2014	187	1818	A
D1A31A1_D1A31A2	410	322	4748	4643	301	167	146	110	A
D1A5KC1_D1A5KC2	411	286	80	5	79	4	61	4	A
D1A7KA1_D1A7KA2	330	349	232	1644	619	134	429	109	A
D1A9XA1_D1A9XA5	361	198	137	414	26	16	21	11	A
D1AK2_1_D1AK2_2	154	352	4225	4290	11	33	24	52	A
D1AMOA1_D1AMOA2	358	685	5565	7345	219	515	241	558	A
D1AMOA1_D1AMOA3	339	685	6785	4966	5	31	6	31	A
D1AQT_1_D1AQT_2	131	374	33	12	6	1	2	1	A
D1AY0A2_D1AY0A3	289	274	58	9	2	1	1	1	A
D1BGLA3_D1BGLA5	462	208	4201	4681	1	1	1	1	A
D1CIY_1_D1CIY_3	357	516	31	149	129	71	145	74	A
D1CLIA1_D1CLIA2	330	245	1436	237	1	1	1	1	A
D1CQIA1_D1CQIA2	254	260	4046	3497	14	3	12	2	A
D1CQXA1_D1CQXA2	234	278	232	204	20	1	22	1	A
D1CQXA2_D1CQXA3	222	278	5924	4210	1	1	1	1	A
D1CT9A1_D1CT9A2	411	246	129	2119	97	67	65	48	A
D1DLIA1_D1DLIA2	280	326	55	3997	54	26	48	20	A
D1DO8A1_D1DO8A2	437	310	2927	2426	1	1	1	1	A
D1E0DA2_D1E0DA3	269	257	1313	735	636	314	675	294	A
D1EE8A1_D1EE8A2	196	303	8234	3821	935	438	900	444	A
D1FC5A1_D1FC5A3	207	236	1498	44	11	3	6	3	A
D1FC5A2_D1FC5A3	289	236	189	178	425	116	302	121	A
D1FJGE1_D1FJGE2	141	387	6	60	1	1	1	1	A
D1FSZ_1_D1FSZ_2	315	326	3656	3298	1	1	1	1	A
D1OT5A1_D1OT5A2	393	253	4683	1720	1	1	1	1	A
D2FFHA2_D2FFHA3	292	334	4280	5708	1290	1877	839	1431	A
D1KRHA1_D1KRHA2	206	330	5976	6457	21	20	2	3	B
D1B23P1_D1B23P3	275	309	110	5981	18	38	16	44	B
D1DAR_3_D1DAR_4	208	260	162	152	57	15	24	7	B
D1BMFA2_D1BMFA3	250	337	24	270	39	32	23	22	B
D1PV4A2_D1PV4A3	241	265	599	4982	228	371	177	267	B
D1A62_1_D1A62_2	118	250	14	2450	4	12	4	4	B
D1C47A3_D1C47A4	227	337	2323	2477	1	1	2	1	B
D1FMTA1_D1FMTA2	273	293	8504	5375	500	810	241	466	B
D1E1CA1_D1E1CA2	642	376	120	1099	6	9	6	9	B
D1A9XA3_D1A9XA5	358	198	1537	2462	1	1	1	1	B
D1BG0_1_D1BG0_2	329	226	4347	2015	1	1	1	1	B
D1AUA_1_D1AUA_2	255	364	50	43	8	2	6	2	B
D1E5TA1_D1E5TA2	498	378	74	81	10	3	5	1	B
D1AY0A2_D1AY0A1	509	274	88	1665	1	1	1	1	B
D1AP5A1_D1AP5A2	190	355	774	2019	1	1	1	1	B
D1AR1B1_D1AR1B2	223	374	107	814	1524	963	1907	1356	B
D1DTWB1_D1DTWB2	299	339	22	4	19	1	6	1	B
D1M6NA1_D1M6NA2	322	293	2056	1863	61	27	61	41	B
D1K7YA2_D1K7YA3	410	225	3148	3161	33	9	38	13	B
D1AIPA1_D1AIPA2	163	306	754	2493	98	100	59	36	B
D1BGXT2_D1BGXT4	537	266	4969	6728	269	409	226	310	B
D1A2OA1_D1A2OA2	307	330	8751	3736	127	92	97	71	B
D1K7YA1_D1K7YA3	372	225	9576	5275	28	27	43	39	B
D1F1HA1_D1F1HA2	365	294	712	168	1	1	2	1	B
D1A3WA2_D1A3WA3	191	296	2037	2694	211	204	431	345	B
D1FGS_1_D1FGS_2	344	298	226	674	249	145	330	210	B
D1E4EA1_D1E4EA2	335	315	3	20	1	1	2	1	C
D1EG9A1_D1EG9A2	373	249	12	472	33	19	21	17	C
D1AH5_1_D1AH5_2	270	278	2924	2876	1	1	1	1	C
D1BIF_1_D1BIF_2	397	296	11	9553	2	1	1	1	C
D1AUP_1_D1AUP_2	363	291	102	6293	28	25	27	24	C
D1FFUC1_D1FFUC2	280	394	15	36	19	2	7	1	C
D1A9XB1_D1A9XB2	326	198	560	361	100	74	113	92	C
D1AZYA1_D1AZYA2	321	199	4491	1858	1	1	1	1	C
D1A0P_1_D1A0P_2	252	278	8987	2709	231	218	149	157	C
D1B70B3_D1B70B6	202	228	6484	5287	793	883	345	440	C
D1BGLA4_D1BGLA5	383	208	129	1229	12	11	4	4	C
D1EE8A1_D1EE8A3	119	303	4145	433	11	7	12	9	C
D1DNPA1_D1DNPA2	444	283	1502	1842	1	1	1	1	C
D1F0YA1_D1F0YA2	288	361	25	7	9	2	9	2	C
D1BGLA1_D1BGLA5	405	208	505	431	24	14	21	13	C
D1EX1A1_D1EX1A2	451	808	939	951	1	1	1	1	C
D1A7AA1_D1A7AA2	335	384	107	7310	13	14	21	21	C
D3R1RA1_D3R1RA2	605	228	7532	7066	4505	4820	3512	3886	C
D1BKHA1_D1BKHA2	355	273	5	223	1	1	1	1	C
D1BGXT1_D1BGXT4	474	266	4131	5953	96	96	152	127	C
D1E9IA1_D1E9IA2	421	253	35	660	1	1	1	1	C
D1O4UA1_D1O4UA2	246	388	8	342	2	1	2	1	C
D1FQVB1_D1FQVB2	136	381	3	218	1	1	1	1	C
D1EUQA1_D1EUQA2	490	243	7219	6189	1	1	1	1	C
D1HX9A1_D1HX9A2	459	365	79	898	1	1	1	1	C
D1IWOA1_D1IWOA4	192	319	185	95	1	1	1	1	C

Table S1: The list of protein complexes in the data set, with the number of amino acid residues in the complex (N), the number of sequences in the MSA (M), the top near native complex prediction for the different scoring functions used in the Main Text, and the test set the protein complexes belonged to.
