# Supplementary material for: Improving protein-protein interaction prediction using evolutionary information from low-quality MSAs
Source: PLoS One. 2017 Feb 6;12(2):e0169356. doi: 10.1371/journal.pone.0169356 (PMC5293240; doi:10.1371/journal.pone.0169356)
Supplement: S2 Table — GREMLIN and EVCOMPLEX predictions for the protein complexes in the data set. For each complex, the number of sequences in the MSA relative to the number of residues in the complex (M/N) and the rank of the top predicted native contact for both methods are shown. In the M/N columns, ‘—’ means no predictions due to not enough sequences (GREMLIN) or failed concatenation (EVCOMPLEX). (PDF) [file pone.0169356.s007.pdf]

complex	M/N GREMLIN	M/N EVCOMPLEX	top native GREMLIN	top native EVCOMPLEX
D1A04A1_D1A04A2	0.45	none	117.140	96
D1A31A1_D1A31A2	0	—	0.02	1245
D1A5KC1_D1A5KC2	0.01	—	0.411	22864
D1A7KA1_D1A7KA2	0.06	—	4.236	39
D1A9XA1_D1A9XA5	0.04	—	3.306	28
D1AK2_1_D1AK2_2	0.01	—	2.467	3317
D1AMOA1_D1AMOA2	0.03	—	1.726	1279
D1AMOA1_D1AMOA3	0.03	—	1.850	1958
D1AQT_1_D1AQT_2	0.01	—	4.496	2149
D1AY0A2_D1AY0A3	0.03	—	15.521	12727
D1BGLA3_D1BGLA5	0.11	24	8.157	34
D1CIY_1_D1CIY_3	0	—	0.618	22863
D1CLIA1_D1CLIA2	0.01	—	4.379	10886
D1CQIA1_D1CQIA2	0.04	—	2.160	287
D1CQXA1_D1CQXA2	0.1	—	3.156	3651
D1CQXA2_D1CQXA3	0.07	—	26.360	4274
D1CT9A1_D1CT9A2	0.12	—	7.733	7401
D1DLIA1_D1DLIA2	0.07	—	9.791	1699
D1DO8A1_D1DO8A2	0.03	—	3.035	3009
D1E0DA2_D1E0DA3	0.51	none	6.548	8373
D1EE8A1_D1EE8A2	0.04	—	9.634	9
D1FC5A1_D1FC5A3	0.04	—	8.672	7265
D1FC5A2_D1FC5A3	0.03	—	8.730	97
D1FJGE1_D1FJGE2	0.03	—	2.562	181
D1FSZ_1_D1FSZ_2	0.03	—	2.384	5
D1OT5A1_D1OT5A2	0.01	—	1.164	315
D2FFHA2_D2FFHA3	0.09	—	2.596	710
D1KRHA1_D1KRHA2	0.08	—	30.676	128
D1B23P1_D1B23P3	0.23	105	4.535	385
D1DAR_3_D1DAR_4	0.03	—	6.155	54
D1BMFA2_D1BMFA3	0.07	—	1.004	14
D1PV4A2_D1PV4A3	0.02	—	0.695	13
D1A62_1_D1A62_2	0.02	—	3.097	56
D1C47A3_D1C47A4	0.01	—	0.565	3167
D1FMTA1_D1FMTA2	0.05	—	7.814	11274
D1E1CA1_D1E1CA2	0.4	2	0.600	9730
D1A9XA3_D1A9XA5	0.04	—	2.332	1842
D1BG0_1_D1BG0_2	0	—	0.031	17
D1AUA_1_D1AUA_2	0.01	—	—	—
D1E5TA1_D1E5TA2	0.03	—	15.078	4545
D1AY0A2_D1AY0A1	1.69	none	8.636	463
D1AP5A1_D1AP5A2	0.04	—	5.928	3270
D1AR1B1_D1AR1B2	0.01	—	5.156	39
D1DTWB1_D1DTWB2	0.05	—	11.927	14047
D1M6NA1_D1M6NA2	0.1	—	4.266	20
D1K7YA2_D1K7YA3	0.02	—	1.724	12003
D1AIPA1_D1AIPA2	0.02	—	1.516	2071
D1BGXT2_D1BGXT4	0.12	none	2.266	37344
D1A2OA1_D1A2OA2	7.65	none	7.265	7
D1K7YA1_D1K7YA3	0.03	—	2.065	322
D1F1HA1_D1F1HA2	0.02	—	3.392	811
D1A3WA2_D1A3WA3	0.22	—	7.362	953
D1FGS_1_D1FGS_2	0.01	—	11.034	77
D1E4EA1_D1E4EA2	0.01	—	6.058	80
D1EG9A1_D1EG9A2	0.01	—	4.229	900
D1AH5_1_D1AH5_2	0.04	—	4.502	2898
D1BIF_1_D1BIF_2	0.01	—	3.548	2953
D1AUP_1_D1AUP_2	0.08	—	4.020	6720
D1FFUC1_D1FFUC2	0.03	—	7.585	9506
D1A9XB1_D1A9XB2	0.06	—	3.320	970
D1AZYA1_D1AZYA2	0.01	—	5.395	128
D1A0P_1_D1A0P_2	0.09	—	24.702	178
D1B70B3_D1B70B6	0	—	6.477	7620
D1BGLA4_D1BGLA5	0.27	1	1.816	1095
D1EE8A1_D1EE8A3	0.01	—	14.614	770
D1DNPA1_D1DNPA2	0.05	—	4.005	2186
D1F0YA1_D1F0YA2	0.05	—	11.964	3446
D1BGLA1_D1BGLA5	0.02	—	7.762	767
D1EX1A1_D1EX1A2	0.1	—	6.029	3237
D1A7AA1_D1A7AA2	0.01	—	1.100	7486
D3R1RA1_D3R1RA2	0.01	—	0.579	4289
D1BKHA1_D1BKHA2	0.11	—	11.634	2160
D1BGXT1_D1BGXT4	0.1	—	2.597	764
D1E9IA1_D1E9IA2	0.05	—	2.004	3410
D1O4UA1_D1O4UA2	0	—	5.744	2861
D1FQVB1_D1FQVB2	0.01	—	—	—
D1EUQA1_D1EUQA2	0.02	—	1.034	42316
D1HX9A1_D1HX9A2	0.04	—	—	—
D1IWOA1_D1IWOA4	0.07	—	14.513	23

Table S2: GREMLIN and EVCOMPLEX predictions for the protein complexes in the data set. For each complex, the number of sequences in the MSA relative to the number of residues in the complex (M/N) and the rank of the top predicted native contact for both methods are shown. In the M/N columns, '—' means no predictions due to not enough sequences (GREMLIN) or failed concatenation (EVCOMPLEX). Predictions were made using the default values of the webservers: for GREMLIN, alignments were generated using HHblits with an E-value of 1E-20 and 8 iterations, and for EVCOMPLEX, an E-value of 1E-5 was used. For none of the complexes there were more than one correctly predicted contacts among the top 20 contact predictions. We note that an exact comparison of these methods and the CMM based method presented here is not directly possible due to differences in the alignment generation.
